# The Effect of Team Census on Outcomes in Trauma Patients

**DOI:** 10.7759/cureus.88027

**Published:** 2025-07-15

**Authors:** Anthony J Duncan, David R Velez, Khaled Zreik

**Affiliations:** 1 Department of Surgery, University of North Dakota School of Medicine and Health Sciences, Fargo, USA; 2 Department of Trauma and Acute Care Surgery, Sanford Health, Fargo, USA

**Keywords:** acute delirium, institutional workload, patient discharge, trauma centers, workload

## Abstract

Background: Physician workload has steadily increased, with higher workloads linked to worse patient outcomes. However, the impact of workload on trauma care remains unexplored. This study aims to evaluate the impact of the census on trauma patients' outcomes.

Methods: We conducted a five-year retrospective analysis of all trauma patients admitted to our Level I trauma center. A multivariate regression analysis was performed to assess the relationship between team census on the day of admission and patient outcomes, adjusting for Injury Severity Score (ISS).

Results: Patients admitted on high census days had significantly higher ISS. After adjusting for ISS, these patients demonstrated significantly higher rates of delirium but no significant differences in other complications. Notably, hospital length of stay was significantly shorter for patients admitted on high census days.

Conclusion: The most severely injured trauma patients often present on the busiest days, potentially straining clinical resources. Higher delirium rates and shorter lengths of stay raise concerns about whether these patients receive optimal care or are being discharged prematurely. These findings underscore the critical need to preserve care quality during high-volume periods and to develop strategies that mitigate the impact of rising workloads on trauma patient outcomes.

## Introduction

With changes in healthcare organisation and delivery, the intensity of physician workload has been gradually increasing [1]. Recent studies have evaluated the negative effects of physician and team census on patient outcomes [2-4]. Within the emergency room, higher census has been associated with increased patient admission rates [3]. Studies in hospitalist groups have shown that increased workload correlates with longer length of stay, higher costs, lower teaching effectiveness, and a greater risk of patient safety events [2,4]. Nearly one-quarter of hospitalists have reported ordering potentially unnecessary tests, procedures, or consultations due to inadequate time for in-person patient evaluations [5]. However, the data remains controversial, as other studies demonstrate minimal differences in resource utilisation with no significant impact on patient outcomes [6].

Within the intensive care unit (ICU), higher ICU occupancy days have been linked to increased rates of premature ICU discharge, although mortality differences remain inconclusive [7-9]. Additionally, higher total hospital occupancy has been associated with significant ICU transfer delays [10]. In pediatric ICUs, rounding teams have disproportionately shortened plan-of-care discussions for low-acuity patients on high-census days, while this effect was not observed in high-acuity patients [11].

At present, no studies have evaluated the effect of team census on outcomes for surgical or trauma patients. This study aims to assess the impact of daily census levels on trauma patients admitted to our institution.

## Materials and methods

Study setting

Our facility is a Level 1 trauma center in the Upper Midwest of the United States. The Trauma and Acute Care Surgery (TRACS) team manages both trauma and emergency general surgery patients. The team is staffed by a rotating faculty of seven fellowship-trained surgical critical care surgeons. The resident team typically consists of a chief resident and two to three junior residents, working in conjunction with one to three nurse practitioners. Provider staffing is not adjusted based on patient census, resulting in significant variability in the patient-to-provider ratio, ranging from one provider for every two patients on low-census days to one provider for as many as fourteen patients during peak times.

Data collection

We conducted a five-year retrospective review of all trauma patients admitted to our center between July 1, 2017, and June 30, 2022. Daily patient census was determined by the number of patients with an active Trauma and Acute Care Surgery order in the electronic medical record system. High and low census groups were then defined by determining the mean census across the 5 years. High and low census days were defined as above or below the average patient census, respectively.

Demographic data included age, sex, and ethnicity. Examined comorbidities included hypertension, diabetes, end-stage renal disease on hemodialysis, cirrhosis, chronic obstructive pulmonary disease (COPD), congestive heart failure, angina, myocardial infarction, peripheral arterial disease, stroke, dementia, bleeding disorders, anticoagulation use, steroid use, chemotherapy, smoking, alcohol abuse, substance abuse, and pregnancy. Patient census was also compared to the mechanism of injury.

Initial presentation data included heart rate, respiratory rate, pulse oximetry, systolic blood pressure, temperature, Glasgow Coma Scale (GCS) [12], and Injury Severity Score (ISS) [13]. The scoring systems are widely adopted in research and clinical settings, and are appropriately cited and not reproduced in full, from the proprietary sources. We evaluated injury patterns, including traumatic brain injury, skull fracture, facial fracture, cervical spine fracture, thoracic/lumbar spine fracture, spinal cord injury, spleen injury, liver injury, kidney injury, pancreas injury, adrenal injury, stomach injury, small intestine injury, colon injury, rectal injury, rib fractures, pulmonary injury, cardiac injury, cerebral vascular injury, thoracic vascular injury, abdominal vascular injury, peripheral vascular injury, upper extremity fracture, lower extremity fracture, and pelvic fracture.

Complications examined included acute kidney injury, infection, pressure ulcer, deep venous thrombosis, pulmonary embolism, cardiopulmonary resuscitation, delirium, unplanned surgery, unplanned intubation, ICU readmission, and withdrawal of care. Outcomes included hospital length of stay, ICU length of stay, ventilator days, and discharge disposition. For regression analysis, complications and outcomes were adjusted for Injury Severity Score.

Statistical analysis

Parametric variables were evaluated using a Student's t-test, while qualitative and nonparametric variables were analysed using a Mann-Whitney U test. A Shapiro-Wilk test confirmed a non-normal distribution for hospital length of stay, ICU length of stay, and ventilator days, with significant right skew. Multivariate linear and logistic regression analyses were performed for complications and outcomes, adjusting for Injury Severity Score. Statistical analyses were conducted using Stata version 16.0 (StataCorp. 2019, Stata Statistical Software: Release 16. College Station, TX: StataCorp LLC). Tests of significance were two-sided, with p-values considered significant at the 0.05 level.

## Results

A total of 4,696 trauma patients were admitted between July 1, 2017, and June 30, 2022. The average daily census was 40.8 patients, ranging from 15 to 70 patients. There were 2,345 patients admitted on low-census days and 2,351 patients admitted on high-census days. From May to October, the average census was 43.8 patients, while from November to April, it was 36.6 patients (p<0.001).

Patient demographics and comorbidities for trauma patients admitted on high-census days were compared to those admitted on low-census days (Table 1). Patients admitted on high-census days had lower rates of hypertension, but no other significant differences were observed.

**Table 1:** Comparison of patient demographics and comorbidities. ^1^mean (SD), t-test; ^2^ :% (n), Chi-Square; *Denotes p-value<0.05.

	Low census (N=2,345)	High census (N=2,351)	p-value
Demographics			
Age (Years)^1^	53.9 (12.4)	53.4 (14.2)	0.364
Male sex^2^	66.7% (1,564)	66.3% (1,559)	0.805
White patient^2^	82.5% (1,934)	81.0% (1,904)	0.188
Black patient^2^	2.6% (61)	3.4% (80)	0.126
American native​​​​​^2^	12.8% (300)	13.3% (313)	0.567
Asian native^2^	0.6% (14)	0.5% (12)	0.428
Comorbidities			
Hypertension^2^	35.7% (837)	32.3% (759)	0.010*
Diabetes^2^	3.6% (319)	12.5% (294)	0.303
Hemodialysis^2^	0.8% (19)	1.0% (24)	0.449
Cirrhosis^2^	1.7% (40)	1.6% (38)	0.640
COPD^2^	6.2% (145)	6.1% (143)	0.934
Congestive heart failure^2^	6.5% (152)	6.2% (146)	0.659
Angina^2^	1.6% (38)	2.4% (56)	0.062
Myocardial infarction^2^	0.6% (14)	0.6% (14)	0.700
Peripheral arterial disease^2^	0.5% (12)	0.8% (19)	0.210
Stroke^2^	2.3% (54)	2.8% (66)	0.273
Dementia^2^	3.8% (89)	3.2% (75)	0.332
Bleeding disorder^2^	52.2% (52)	2.9% (68)	0.118
Anticoagulation^2^	13.7% (321)	13.1% (308)	0.584
Steroid use^2^	2.7% (63)	2.3% (54)	0.447
Chemotherapy^2^	1.0% (23)	1.1% (26)	0.576
Smoker^2^	47.3% (1,109)	47.7% (1,121)	0.326
Alcohol abuse^2^	10.7% (251)	10.9% (256)	0.838
Substance abuse^2^	10.2% (239)	10.3% (242)	0.909
Pregnant^2^	0.2% (5)	0.2% (5)	0.997

Mechanisms of injury for trauma patients admitted on high-census days were compared to those admitted on low-census days (Table 2). Patients admitted on high-census days were significantly more likely to have sustained a motorcycle crash, bicycle crash, or watersports-related trauma, whereas falls and snowmobile crashes were more common on low-census days.

**Table 2:** Mechanism of injury. ^1^ : % (n), Chi-square; *denotes p-value<0.05 ATV: All-terrain vehicle.

Mechanism	Low census (N=2,345)	High census (N=2,351)	p-value
Fall^1^	44.1% (1,034)	40.4% (950)	0.011*
Motor vehicle crash^1^	21.3% (499)	21.9% (515)	0.627
Motorcycle crash^1^	2.9% (68)	5.6% (132)	<0.001*
ATV crash^1^	2.9% (68)	3.7% (87)	0.145
Snowmobile crash^1^	1.5% (35)	0.3% (7)	<0.001*
Bicycle crash^1^	1.0% (23)	2.0% (47)	0.005*
Pedestrian trauma^1^	2.7% (63)	2.6% (61)	0.775
Sport trauma^1^	0.4% (9)	0.2% (5)	0.164
Watersport trauma^1^	0.0% (0)	0.5% (12)	0.002*
Machining trauma^1^	1.3% (30)	1.2% (28)	0.787
Animal trauma^1^	0.6% (14)	0.6% (14)	0.858
Hanging^1^	0.5% (12)	0.8% (19)	0.145
Assault^1^	5.3% (124)	4.9% (115)	0.537
Gunshot wound^1^	3.1% (73)	2.4% (56)	0.151
Stab wound^1^	3.8% (85)	3.4% (80)	0.425
Fireworks^1^	3.6% (84)	5.0% (118)	0.319
Burn^1^	2.0% (47)	2.0% (47)	0.908

Presentation and injury patterns for trauma patients admitted on high-census days were compared to those on low-census days (Table 3). Patients admitted on high-census days had significantly higher initial heart rates, temperatures, and Injury Severity Scores. These patients also had significantly higher rates of rib fractures and pulmonary injuries, though no other significant injury pattern differences were noted.

**Table 3:** Presentation and injuries. ^1^mean (SD), t-test; ^2^: % (n), Chi-Square; *denotes p-value <0.05.

	Low census (N=2,345)	High census (N=2,351)	p-value
Presentation			
Heart rate (bpm)^1^	86 (10)	88 (12)	0.021*
Respiratory rate (bpm)^1^	18 (2)	18 (3)	0.178
Pulse oximetry^1^	96% (1)	96% (1)	0.361
Systolic blood pressure (mmHg)^ 1^	136 (9)	136 (13)	0.855
Temperature (°C)^1^	36.7 (0.3)	36.8 ( 0.5)	0.013*
Glasgow coma scale (GCS)^1^	13.7 (3)	13.6 (3)	0.533
Injury severity score (ISS)^ 1^	11.5 (4)	12.3 ( 7)	0.007*
Injuries			
Traumatic brain injury (TBI)^2^	32.1% (752)	33.0% (776)	0.512
Skull fracture^2^	9.3% (218)	9.6% (226)	0.710
Facial fracture^2^	13.7% (321)	15.1% (355)	0.196
C-Spine fracture^2^	11.9% (279)	12.4% (291)	0.553
T/L-Spine fracture^2^	19.0% (446)	20.9% (492)	0.102
Spinal cord injury^2^	4.2% (98)	4.1% (96)	0.869
Spleen^2^	3.1% (73)	3.6% (85)	0.258
Liver^2^	2.8% (66)	3.1% (73)	0.381
Kidney^2^	1.7% (40)	1.6% (38)	0.625
Pancreas^2^	0.2% (5)	0.2% (5)	0.997
Adrenal^2^	0.7% (16)	0.9% (21)	0.520
Stomach^2^	0.3% (7)	0.3% (7)	0.996
Duodenum/small intestine^2^	1.0% (23)	0.9% (21)	0.624
Colon^2^	0.9% (21)	1.1% (26)	0.870
Rectum^2^	0.1% (2)	0.2% (5)	0.258
Rib fracture^2^	26.7% (626)	29.6% (695)	0.020*
Pulmonary^2^	15.0% (352)	18.0% (423)	0.005*
Cardiac^2^	0.6% (14)	0.8% (19)	0.496
Cerebral vascular injury^2^	1.8% (42)	2.0% (47)	0.758
Thoracic vascular injury^2^	0.5% (12)	0.8% (19)	0.145
Abdominal vascular injury^2^	0.5% (12)	0.9% (21)	0.087
Peripheral vascular injury^2^	1.1% (263)	1.4% (33)	0.364
Upper extremity fracture^2^	11.3% (265)	10.7% (251)	0.494
Lower extremity fracture^2^	14.2% (333)	15.3% (360)	0.302
Pelvic fracture^2^	9.4% (220)	9.2% (216)	0.819

Complications (Table 4) and outcomes (Tables 5-6) were analysed while adjusting for ISS. Patients admitted on high-census days had a significantly higher risk of developing delirium but exhibited no other differences in complications. Additionally, these patients had significantly shorter hospital stays and were more likely to be discharged with home health services or to a rehabilitation facility, but were less likely to be discharged to a skilled nursing facility.

**Table 4:** Patient complications adjusted by Injury Severity Score. *Denotes p<0.05

	Low census (N=2,345)	High census (N=2,351)	Adjusted odds ratio (95% Confidence interval)	p-value
Any complication	9.0%	8.4%	0.85 (0.69-1.05)	0.144
Acute kidney injury	0.7%	0.7%	0.93 (0.47-1.84)	0.838
Infection	0.8%	0.9%C	1.16 (0.60-2.21)	0.662
Pressure ulcer	0.7%	0.7%	0.93 (0.46-1.87)	0.835
Deep venous thrombosis	1.1%	1.4%	1.24 (0.73-2.11)	0.421
Pulmonary embolism	0.7%	0.6%	0.84 (0.42-1.69)	0.623
Cardiopulmonary resuscitation	0.7%	0.7%	0.93 (0.47-1.84)	0.839
Delirium	1.0%	2.2%	2.05 (1.25-3.39)	0.005*
Unplanned surgery	0.8%	0.9%	1.09 (0.59-2.03)	0.788
Unplanned intubation	1.9%	1.7%	0.85 (0.55-1.32)	0.474
ICU readmission	2.0%	1.7%	0.83 (0.54-1.27)	0.385
Withdrawal	1.1%	1.1%	0.99 (0.57-1.70)	0.960

**Table 5:** Patient outcomes adjusted by Injury Severity Score (coefficient). *Denotes p-value <0.05.

	Low census (N=2,345)	High census (N=2,351)	Coefficient (95% Confidence interval)	p-value
Hospital length of stay (days)	4	3	-0.42 (-0.82 to -0.01)	0.044*
ICU length of stay (days)	3	3	-0.23 (-0.70 to 0.24)	0.337
Ventilator days (days)	2	2	-0.36 (-1.43 to 0.71)	0.510

**Table 6:** Patient outcomes adjusted by Injury Severity Score (adjusted odds ratio). *Denotes p-value <0.05.

	Low census (N=2,345)	High census (N=2,351)	Adjusted odds ratio (95% confidence interval)	p-value
Discharge disposition				
Home	55.90%	54.40%	0.98 (0.87-1.10)	0.751
Home health	4.10%	6.00%	1.50 (1.15-1.96)	0.003*
Rehab	9.50%	11.50%	1.22 (1.02-1.44)	0.044*
Nursing home	12.50%	10.50%	0.83 (0.69-0.98)	0.038*
Swing bed	2.00%	2.60%	1.24 (0.84-1.82)	0.275
Long-term acute care	2.60%	2.20%	0.75 (0.51-1.10)	0.136
Inpatient psychiatry	1.40%	1.40%	1.04 (0.64-1.69)	0.876
Death	4.40%	4.30%	0.91 (0.68-1.22)	0.538
Hospice	0.60%	0.60%	1.78 (0.88-3.61)	0.109
Against medical advice	1.50%	1.20%	0.86 (0.53-1.41)	0.55

## Discussion

This study aimed to evaluate trauma patient outcomes based on average daily service census, with days categorised as high or low census depending on whether the patient load was above or below the overall average. We also find that our facility experienced a notable increase in patient census from May to October compared to November to April, with a 20% higher census during summer months. This likely correlates with increased trauma incidence due to favorable road conditions and an influx of vacationers. Weather fluctuations, including significant snowfall and extreme low temperatures, may account for seasonal variations in injury mechanisms [14,15]. 

Patients admitted on high-census days had lower rates of hypertension but showed no other significant demographic or comorbidity differences. They were more likely to suffer motorcycle, bicycle, and watersports-related injuries, while falls and snowmobile crashes were more prevalent on low-census days.

Patients presenting on high census days exhibited higher initial heart rates and temperatures, while other vital signs showed no significant differences. They also had slightly higher Injury Severity Scores and were more likely to sustain rib fractures and pulmonary trauma, though other injury patterns remained comparable. This suggests that patients presenting on high census days may have different clinical needs compared to those presenting on low census days. Proper planning and resource allocation are essential to ensure adequate coverage and optimal care for these patients.

After controlling for Injury Severity Scores, morbidity and mortality were not significantly different; patients admitted on high-census days had more than double the odds of developing delirium (2.2% on high census days vs 1.0% on low census days). While the exact cause of increased delirium cannot be determined from this study, there are plausible explanations. Our previous study also demonstrated increased delirium rates in traumatic brain injury patients admitted on high-census days [16]. If patients are receiving less attention and care due to an increased workload or high census, it may, to some extent, account for an increased risk for delirium. Patient groups had comparable prehospital comorbidities, including substance and alcohol use; however, unaccounted variables may still influence the observed rates of delirium. Regardless of the underlying cause, delirium is associated with prolonged length of stay and worse clinical outcomes, underscoring the importance of prevention as a consistent priority for providers [17].

Shorter hospital stays and altered discharge dispositions suggest potential pressure to expedite discharges. This is exhibited by the notable finding that patients admitted on high census days had a shorter length of stay, even when controlling for Injury Severity Scores. This was somewhat counterintuitive, as one might expect that a higher patient volume and increased workload would lead to longer hospitalisations. One possible explanation is a heightened urgency to expedite patient disposition when capacity is strained. This is reflected in the discharge patterns observed: patients admitted on high census days were more likely to be discharged with home health services or to rehabilitation facilities, which typically have fewer barriers to acceptance. Conversely, they were less likely to be discharged to a nursing home, where placement is often more challenging. Further investigation into readmission rates for these patients could help determine whether they were being discharged prematurely.

Limitations

This review has several limitations. First, it focuses on a single cohort at a Level I trauma center with a broad catchment area. The relationship between team census and patient outcomes is likely context-dependent and may not be generalizable to other settings. Second, at our institution, trauma patients are managed by the trauma and acute care surgery team, which also oversees acute care surgery patients. Institutions where these patient groups are cared for by separate teams may observe different outcomes. Third, our review identified lower complication rates than those typically reported in the literature, potentially due to underreporting within our system. Although the retrospective design limits data accuracy, we find no evidence to suggest this was the case in our analysis. Finally, while we accounted for a comprehensive set of patient-related variables, we were unable to control for facility-specific factors such as fluctuations in nursing and ancillary staff coverage, which may have influenced outcomes.

## Conclusions

With rising physician workload, monitoring its impact on patient outcomes is crucial. At our institution, patients admitted on high-census days had slightly increased ISS and different mechanisms of injury compared to low-census counterparts. Patients admitted on high-census days, however, experienced significantly higher rates of delirium. This finding raises important considerations about whether additional resources should be allocated during periods of high census to mitigate delirium risk. Additionally, these patients had shorter hospital stays and altered discharge patterns, warranting further analysis of long-term outcomes. All other measures of morbidity and mortality were similar between groups. Organisations must ensure quality care despite increasing workloads and develop strategies to manage high-volume periods effectively. Institutions should evaluate resource allocation and availability for patients presenting on high census days.
